# A highly efficient β-glucosidase from the buffalo rumen fungus *Neocallimastix patriciarum* W5

**DOI:** 10.1186/1754-6834-5-24

**Published:** 2012-04-19

**Authors:** Hsin-Liang Chen, Yo-Chia Chen, Mei-Yeh Jade Lu, Jui-Jen Chang, Hiaow-Ting Christine Wang, Huei-Mien Ke, Tzi-Yuan Wang, Sz-Kai Ruan, Tao-Yuan Wang, Kuo-Yen Hung, Hsing-Yi Cho, Wan-Ting Lin, Ming-Che Shih, Wen-Hsiung Li

**Affiliations:** 1 Biodiversity Research Center, Academia Sinica, Taipei, 115, Taiwan; 2 Department of Biological Science & Technology, National Pingtung University of Science & Technology, Neipu Hsiang, Pingtung, 91201, Taiwan; 3 Genomics Research Center, Academia Sinica, Taipei, 115, Taiwan; 4 Molecular and Biological Agricultural Sciences Program, Taiwan International Graduate Program, National Chung-Hsing University – Academia Sinica, Taipei, 115, Taiwan; 5 Graduate Institute of Biotechnology, National Chung-Hsing University, Taichung, 402, Taiwan; 6 Agricultural Biotechnology Research Center, Academia Sinica, Taipei, 115, Taiwan; 7 Program in Microbial Genomics, National Chung-Hsing University, Taichung, 402, Taiwan; 8 Biotechnology Center, National Chung-Hsing University, Taichung, 402, Taiwan; 9 Department of Ecology and Evolution, University of Chicago, Chicago, IL, 60637, USA

**Keywords:** Endoglucanase, β-glucosidase, *Neocallimastix patriciarum*, Rumen fungi, Simultaneous saccharification and fermentation

## Abstract

****Background**:**

Cellulose, which is the most abundant renewable biomass on earth, is a potential bio-resource of alternative energy. The hydrolysis of plant polysaccharides is catalyzed by microbial cellulases, including endo-β-1,4-glucanases, cellobiohydrolases, cellodextrinases, and β-glucosidases. Converting cellobiose by β-glucosidases is the key factor for reducing cellobiose inhibition and enhancing the efficiency of cellulolytic enzymes for cellulosic ethanol production.

****Results**:**

In this study, a cDNA encoding β-glucosidase was isolated from the buffalo rumen fungus *Neocallimastix patriciarum* W5 and is named NpaBGS*.* It has a length of 2,331 bp with an open reading frame coding for a protein of 776 amino acid residues, corresponding to a theoretical molecular mass of 85.1 kDa and isoelectric point of 4.4. Two GH3 catalytic domains were found at the N and C terminals of NpaBGS by sequence analysis. The cDNA was expressed in *Pichia pastoris* and after protein purification, the enzyme displayed a specific activity of 34.5 U/mg against cellobiose as the substrate. Enzymatic assays showed that NpaBGS was active on short cello-oligosaccharides from various substrates. A weak activity in carboxymethyl cellulose (CMC) digestion indicated that the enzyme might also have the function of an endoglucanase. The optimal activity was detected at 40°C and pH 5 ~ 6, showing that the enzyme prefers a weak acid condition. Moreover, its activity could be enhanced at 50°C by adding Mg^2+^ or Mn^2+^ ions. Interestingly, in simultaneous saccharification and fermentation (SSF) experiments using *Saccharomyces cerevisiae* BY4741 or *Kluyveromyces marxianus* KY3 as the fermentation yeast, NpaBGS showed advantages in cell growth, glucose production, and ethanol production over the commercial enzyme Novo 188. Moreover, we showed that the KY3 strain engineered with the NpaNGS gene can utilize 2 % dry napiergrass as the sole carbon source to produce 3.32 mg/ml ethanol when Celluclast 1.5 L was added to the SSF system.

**Conclusion:**

Our characterizations of the novel β-glucosidase NpaBGS revealed that it has a preference of weak acidity for optimal yeast fermentation and an optimal temperature of ~40°C. Since NpaBGS performs better than Novo 188 under the living conditions of fermentation yeasts, it has the potential to be a suitable enzyme for SSF.

## **Background**

Cellulose is the major component in the plant cell wall. It is a linear polymer of polysaccharide consisting of D-glucose units linked by 1,4-β-D-glucosidic bonds. The hydrolysis of plant polysaccharides is catalyzed by microbial cellulases, including endo-β-1,4-glucanases, cellobiohydrolases, cellodextrinases and β-glucosidases [1]. These cellulases are very important in various applications, including saccharification of industrial and agricultural cellulose containing residues, treating cellulose pulp wastes in the paper industry and enhancing the extraction of fermentable substances in the beer brewing and alcohol fermentation industries [2-4]. In the process of cellulose degradation, various endo-β-1,4-glucanases act randomly along the cellulose chains, producing cellulose fragments to generate new sites on which cellodextrinases act to produce cellobiose or oligosaccharides. β-glucosidases then catalyze the hydrolysis of cellobiose and oligosaccharides, which are strong inhibitors of both endo-β-1,4-glucanases and cellodextrinases [5-7]. Thus, converting cellobiose or oligosaccharides to glucose is the key factor for reducing cellobiose inhibition and enhancing the efficient of cellulolytic enzymes [5,8,9].

According to the classification by [10,11], β-glucosidase genes are typically placed into glycosyl hydrolase families 1 and 3 (*GH1* and *GH3*). Generally, the β-glucosidases of bacterial, plant, and mammalian origins that belong to *GH1* usually possess a significant level of galactosidase activity in addition to β-glucosidase activity. The other β-glucosidases of fungi, bacteria, and plants are often classified into *GH3*. The β-glucosidases in both families are known as retaining enzymes because their products retain the same anomeric configuration as the substrate [12]. Their reactions are thought to follow a double-displacement mechanism [12].

Cellulolytic enzymes from brown- and white-rot fungi have been extensively studied in organisms such as *Trichoderma reesei* and *Phanerochaete chrysosporium*[13-16]. It is also known that rumen fungi are able to degrade the most resistant plant cell-wall polymers and therefore are rich sources of fibrolytic enzymes with tremendous potential for industrial and agricultural applications [16-18]. Recently, we reported the transcriptomic and secretomic profiles of the rumen fungus *Neocallimastix patriciarum* W5 [19]. From the transcriptome, we cloned and expressed a cDNA encoding a β-glucosidase. We call this enzyme NpaBGS and have pursued a detailed characterization in the present study. The protein sequence contains two *GH3* domains at the N and C terminals from a domain prediction analysis. In a heterologous expression system, the purified β-glucosidase showed the optimal activity at pH 6 and 40°C and could be significantly enhanced at 50°C by adding Mg^2+^ or Mn^2+^ ions. Furthermore, two fermentation yeasts were chosen to assess the potential of NpaBGS for the SSF process.

## **Results and discussion**

### **Characterization of the β-glucosidase NpaBGS**

The cloned NpaBGS cDNA contains 2,331 bp and the deduced amino acid sequence has 776 amino acid residues, with a theoretical molecular mass of 85.1 kDa and isoelectric point of 4.4 (Additional file 1). The cleavage site for the putative signal peptide is located between residues Ala20 and Ile21. Three potential *N*-glycosylation sites were predicted at residues 51, 223 and 532. Conserved domain prediction suggests that it is a β-glucosidase of the glycosyl hydrolase Family 3 (*GH3*) carrying a *GH3* N-terminal domain (Pfam00933) and a *GH3* C-terminal domain (Pfam01915) located at residues 62 ~ 270 and 350 ~ 577, respectively.

Based on amino acid sequences, glucosidases have been classified into several families, with most of the β-glucosidases belonging to either *GH1* or *GH3*[10,11,20]. Comparative analyses using BlastX and ExPASy indicated that NpaBGS contains two conserved putative domains of *GH3*. The aspartic acid residue Asp251 in the conserved domain (GXVMXD) might be the active-site residue of NpaBGS [21-23].

### **Purification of NpaBGS expressed in*Pichia pastoris***

**Figure 1:**
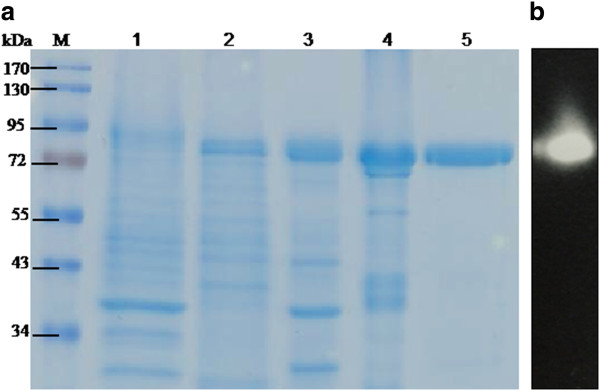
**SDS-PAGE and Zymogram of NpaBGS, which was expressed and purified from a*Pichia pastoris*recombinant strain.** (A) SDS-PAGE of NpaBGS after each purification step. Lane M, protein marker; lane 1, crude extract; lane 2, condensation; lane 3, ammonium sulfate precipitation; lane 4, 1^st^ DEAE; lane 5, 2^nd^ DEAE. (B) Zymogram of the purified β-glucosidase in the native PAGE with MUC substrate staining.

The expression of the NpaBGS cDNA in *P. pastoris* GS115 was conducted under the control of the inducible promoter AOX1 on the pPICZ A vector. A 2-day culture in a medium containing 0.5 % methanol resulted in optimal enzyme production. These conditions were used in subsequent large scale cultures for enzyme purification and the results are summarized in Table 1. The pattern of fractions containing NpaBGS activities at each purification step was checked by SDS-PAGE (Figure 1A). A significant mass of the purified enzyme was estimated to be 85 kDa by SDS-PAGE analysis. The enzyme was purified 2.9-fold with a specific activity of 34.5 U/mg against cellobiose as the substrate. In addition, the β-glucosidase activity of the purified NpaBGS was examined by the zymogram assay with 4-methylumbelliferyl-β-D-cellobioside (MUC) staining after electrophoresis on the native PAGE (Figure 1B).

**Table 1:** Summary of stepwise purification of NpaBGS (β-glucosidase)

**Purification steps**	**Total activity (U)**	**Total protein (mg)**	**Specific activity (U/mg)**	**Purification (fold)**	**Recover rate (%)**
**Supernatant**	2396	198	12.1	1	**100**
**Condensation**	1900	96	19.8	1.6	**79**
**Ammonium sulfate precipitation**	985	47	21.0	**1.7**	**41**
**1**^**st**^**DEAE-Sepharose chromatography**	524	30	17.5	**1.5**	**22**
**2**^**nd**^**DEAE-Sepharose chromatography**	345	10	34.5	**2.9**	**14**

The assay was conducted at 40°C, pH 6 for 1 hr. One unit enzyme activity was defined as 1 nmole of glucose released per minute.

The evidence from enzymatic assay, bioinformatics and homology modeling studies also strongly suggest that NpaBGS is a member of *GH3*. In NpaBGS, three putative *N*-glycosylation sites were found (Additional file 1), but no significant difference was observed between the calculated and the apparent molecular weight on SDS–PAGE using the *Pichia* expression system (Figure 1). Unlike reported cases of glycosylated β-glycosidases [12,24], there might be no or little glycosylation on NpaBGS. Furthermore, NpaBGS has also been successfully expressed in *Escherichia coli, Bacillus subtilisKluyveromyces marxianus* and *K. lactis* (data not shown). The enzyme with no or little glycosylation would be easier to express in both eukaryotic and prokaryotic systems, except for species such as *S. cerevisiae* that have a strong glycosylation mechanism. It will be interesting to do site-directed mutagenesis to study whether any of the three sites are actually glycosylated.

**Figure 2:**
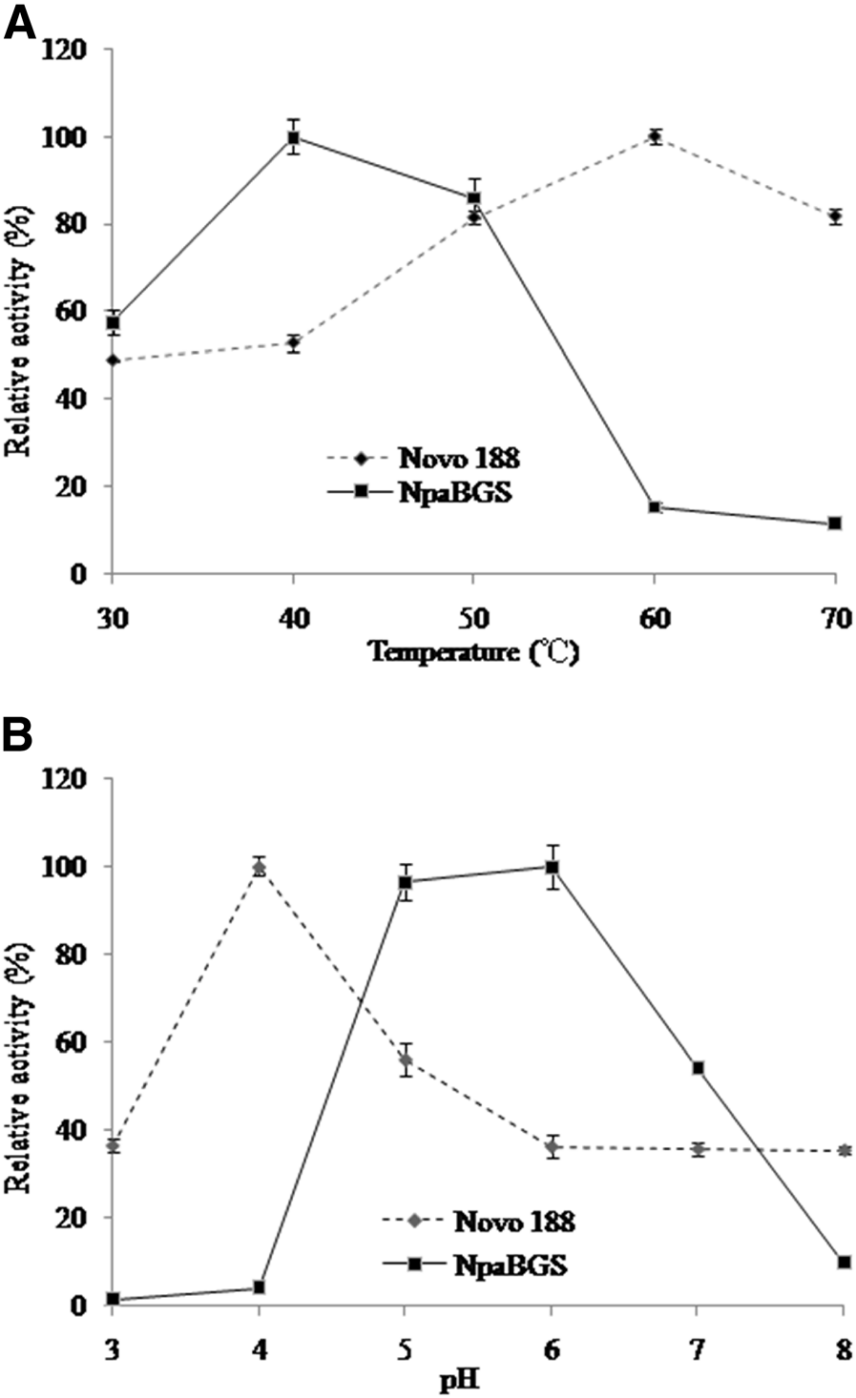
**Effects of temperature (A) and pH (B) on the activity of purified NpaBGS and Novo 188.** The relative activities are expressed as percentage normalized to the sample with the highest activity in each test. The profile of NpaBGS was shown with a solid line, and Novo 188 with a dotted line.

The purified NpaBGS and commercial enzyme Novo 188 were used to evaluate the effects of pH and temperature on enzymatic activity using cellobiose as the substrate. The maximum activities of NpaBGS and Novo 188 were observed at 40°C and 60°C, respectively, and NpaNGS was found to display a higher activity than Novo 188 at 40°C, though a lower activity at 60°C (Figure 2A). Both enzymes showed about 80 % residual activity at 50°C. At pH 6.0, NpaBGS showed a relative activity of 58 % at 30°C and 86 % at 50°C. The effect of pH on the hydrolysis rate was also evaluated for NpaBGS in a reaction system incubated at 40°C for 1 h. High levels of NpaBGS activity were found in a narrow pH range (5.0-6.0), peaking at pH 6.0 (Figure 2B). At pH values outside the range of 5.0 to 8.0, the relative activities decreased significantly. In contrast, Novo 188 showed an acidity-tolerance at pH 4. The activities of NpaBGS under conditions at different pH values and temperature indicated that NpaBGS prefers weak acidity as the yeast fermentation condition.

### **Substrate specificity of NpaBGS**

The NpaBGS substrate specificity was determined using the purified protein to avoid the background β-glucosidase activity of *P. pastoris*. All substrates were assayed at 40°C and pH 6 for 1 h. As shown in Table 2, enzyme activities were detected for natural substrates such as amygdalin, arbutin, larminarin, phenyl-β-D-glucoside, β-gentiobiose as well as cellobiose. It showed little or no activity for other sugars such as sinigrin, maltose, lactose and sucrose. For synthetic substrates, MUC and MUD were found to have the best activity, but no activity was detectable for MUG (data not shown). Moreover, when MUC was used, the enzymatic product generated measurable fluorescence, indicating that this enzyme was active on short cello-oligosaccharides (Figure 1B).

**Table 2:** Substrate specificity of purified NpaBGS

Substrate	Relative activity (%)
Amygdalin	92
Sinigrin	1
Esculin	87
Phenyl-β-D-glucoside	99
Arbutin	95
β-methyl-D-glucoside	11
Cellobiose	100
β-Gentiobiose	93
Maltose	0
Lactose	0
Sucrose	0
Laminarin	98
4-methylumbelliferyl-β-D-cellobioside	265
4-methylumbelliferyl-β-D-glucopyranoside	255
4-mehtylumbelliferyl-β-D- galactopyronoside	0

The assay was performed at 40°C, pH 6 for 1 hr using 0.01units/ml NpaBGS enzyme. The relative activities are expressed as percentage by normalizing to the cellobiose reaction activity.

Most β-glucosidases can be divided into three groups with respect to their substrate specificity: (a) those that exhibit a high specificity towards aryl β-D-glucosides, (b) those that preferentially hydrolyze cellobiose and cello-oligosaccharides (also known as cellobiases), and (c) those that hydrolyze both types of substrates (i.e., broad-specificity β-glucosidases) [24]. From our experiments, NpaBGS displayed a higher activity on 4-methylumbelliferyl substrates than on cellobiose, demonstrating that NpaBGS has a very high affinity for methylumbelliferyl substrates (Table 2). In addition, NpaBGS hydrolyses CMC (Additional file 2) and MUC (Table 2), suggesting that the enzyme possesses both endo-glucanase and β-glucosidase activities [25-28]. Thus, this enzyme showed a strong β-glucosidase activity and might also possess other cellulase functions.

### **Effects of different elements on the NpaBGS activity**

To study the effects of metal ions and reducing agents on the NpaBGS activity, we conducted assays in the presence of metal cations, including Al^3+^, Ca^2+^, Cu^2+^, Fe^3+^, Mg^2+^, Mn^2+^ and Zn^2+^, and reducing agents, such as DTT and β-mercaptoenthanol, at the concentrations of 1 and 10 mM (Table 3). At the concentration of 1 mM, only Mg^2+^, Mn^2+^ and Zn^2+^ showed significant enhancement of β-glucosidase activity compared to the reaction containing the chealator EDTA. When the concentration was increased to 10 mM, Al^3+^, Cu^2+^ and Fe^2+^ all showed significant inhibition of enzymatic activity; Al^3+^ and Cu^2+^ almost abolished the function. In contrast, Ca^2+^, Mg^2+^, Mn^2+^ and Zn^2+^ showed significant enhancement of β-glucosidase activity, especially for Mg^2+^ and Mn^2+^, which showed very strong enhancement (Table 3). Supplementing either DTT or β-mercaptoenthanol showed no apparent effects on enzymatic activity under the condition tested.

**Table 3:** Effect of metal ions and reducing agents on the activity of purified NpaBGS

Metal	1 mM	10 mM
AlCl_3_.6H_2_O	104	7
MgSO_4_.7H_2_O	132	152
CaCl_2_.2 H_2_O	109	136
MgCl_2_.6 H_2_O	132	150
MnCl_2_.2 H_2_O	142	151
ZnCl_2_	116	133
CuCl_2_.2 H_2_O	97	3
FeCl_3_.2 H_2_O	95	37
β-mercaptoenthanol	104	105
Dithiothreitol	103	101
EDTA	100	91
control (NpaBGS at 1:100)	100	100

The assay was performed at 40°C, pH 6 for 1 hr using 0.01units/ml of the NpaBGS enzyme. The relative activities are expressed as percentage by normalizing to the control activity.

**Figure 3:**
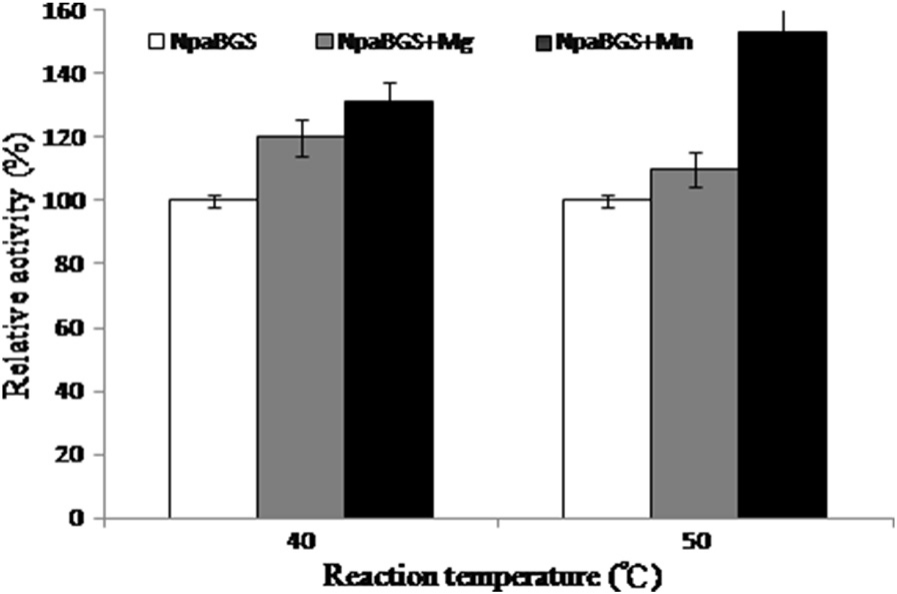
**The effects of Mg**^**2+**^**and Mn**^**2+**^**cations, to a final concentration of 1 and 10 mM, on the activity of purified NpaBGS at pH 6.0.** The activity of NpaBGS (open square), NpaBGS with Mg^2+^ (gray square) and NpaBGS with Mn^2+^ (black square) were compared.

We examined whether Mg^2+^ and Mn^2+^ could enhance pH or temperature tolerance of NpaBGS. We found no effect of these ions on pH tolerance (data not shown), but addition of either cation increased NpaBGS’s activity at higher temperatures (Figure 3). Interestingly, significant enhancement of the activity by the supplement of Mn^2+^ was found at 50°C over those assayed at 40°C, suggesting the potential of *in vitro* application in digesting cellulose at elevated temperatures by supplementing enhancing cations.

According to previous studies, supplementing divalent or trivalent cations is one strategy to increase enzyme reaction efficiency, thermostability or termination reaction. Different levels of inhibitory effect on enzyme activities by metal ions have been reported, especially Cu^2+^ and Fe^2+^ for β-glucosidase [24,29-31]. In a previous study, Ca^2+^ showed several benefits including protein conformation stabilization, higher affinity for the substrate, and a higher thermostability of an endoglucanase of *Clostridium thermocellum*[32]. However, Mg^2+^ and especially Mn^2+^ showed a stronger enhancement than Ca^2+^ on the β-glucosidase activity of NpaBGS (Table 3 & Figure 3). In our tests, Al^3+^, Cu^2+^ and Fe^3+^ showed a significant inhibition of NpaBGS activity at 10 mM (Table 3). This indicated that the requirement of free sulfhydryl groups in NpaBGS is similar to those observed in other β-glucosidases [33]. The sequence data also suggested that there are thiol groups presented at the active site that are involved in binding or catalysis, or that such groups are essential for maintaining a proper tertiary structure of the enzyme [34-36]. This is consistent with the suggestion that the cysteine residue is involved in the stability and activity of β-glucosidases [37]. On the other hand, we found the stimulatory effect of ions on NpaBGS (Table 3) as reported in other studies [28,38]. This stimulation has been regarded as promoting a significant reduction in the binding specificity and/or deactivation of the site [39].

### **Performance of NpaBGS in Simultaneous Saccharification and Fermentation**

**Figure 4:**
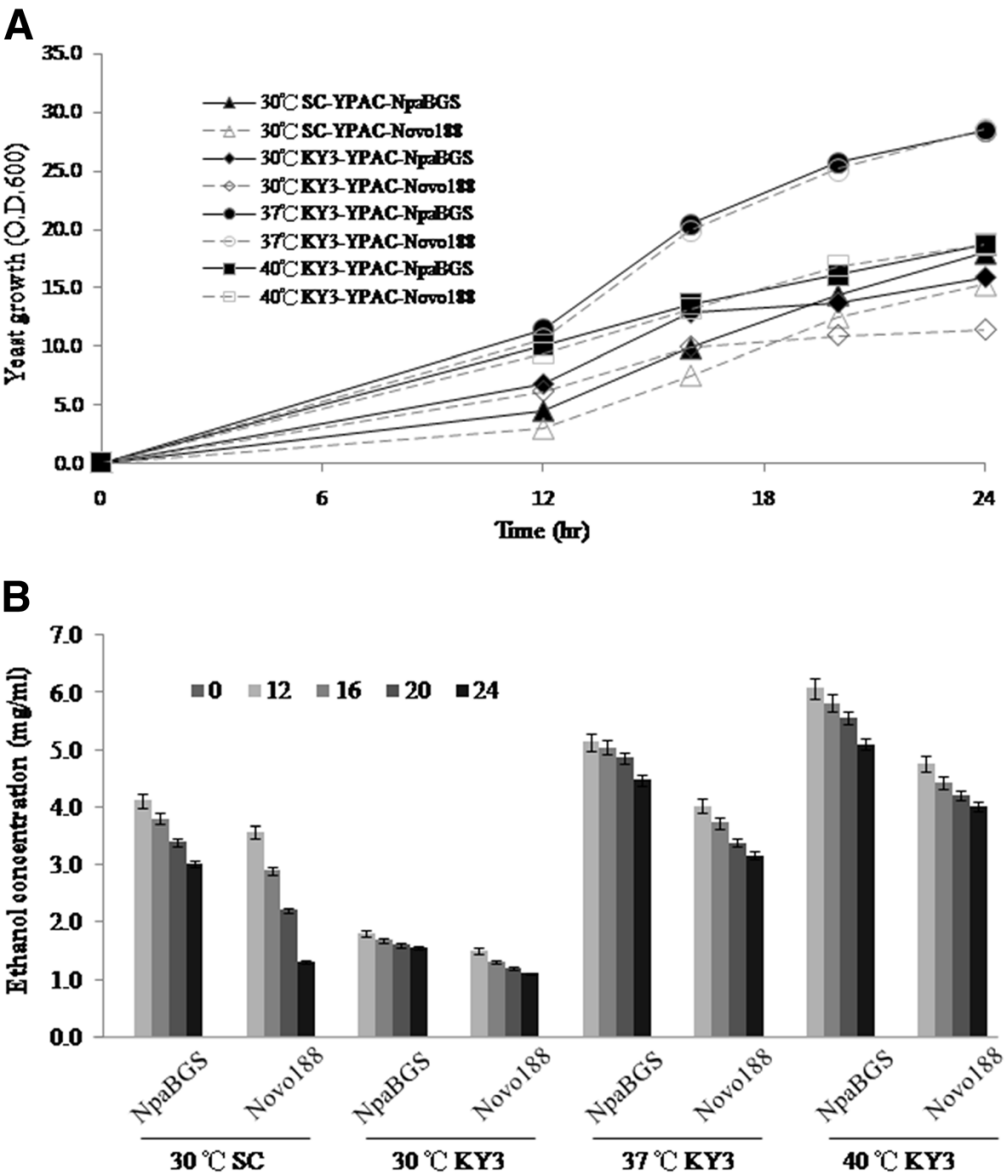
**The performances of NpaBGS and Novo 188 in SSF at different temperatures.** Equal units (2 units) of the two enzymes were added separately to 10 ml yeast cultures with 2 % cellobiose as the sole carbon source and their effects on yeast growth were compared. (A) The cell density assay of growth curve. (B) The ethanol productivity of SSF. SC: *S. cerevisiae* BY4741; KY3*: K. marxianus* KY3.

Since cellobiose digestion by β-glucosidase might be affected by the end-product feedback inhibition, it is not easy to compare the hydrolytic efficiencies of NpaBGS and Novo 188 (data not shown). We therefore employed a SSF process to compare the efficiencies of NpaBGS and Novo 188. We added an equal amount (2 units) of NpaBGS or Novo 188 separately to 10 ml of yeast cultures with 2 % cellobiose as the sole carbon source and compared their effects on yeast growth. The brewers' yeast *Saccharomyces cerevisiae* BY4741 and the thermo-stable kefir yeast *K. marxianus* KY3 [40] were employed in separate experiments. Both cultures showed a better growth profile in cell density with the addition of purified NpaBGS than with the addition of Novo 188 at 30°C (Figure 4A). *K. marxianus* KY3 was also studied at higher temperatures, i.e., 37°C and 40°C. It grew better at 37°C than at 40°C or 30°C (Figure 4A).

**Figure 5:**
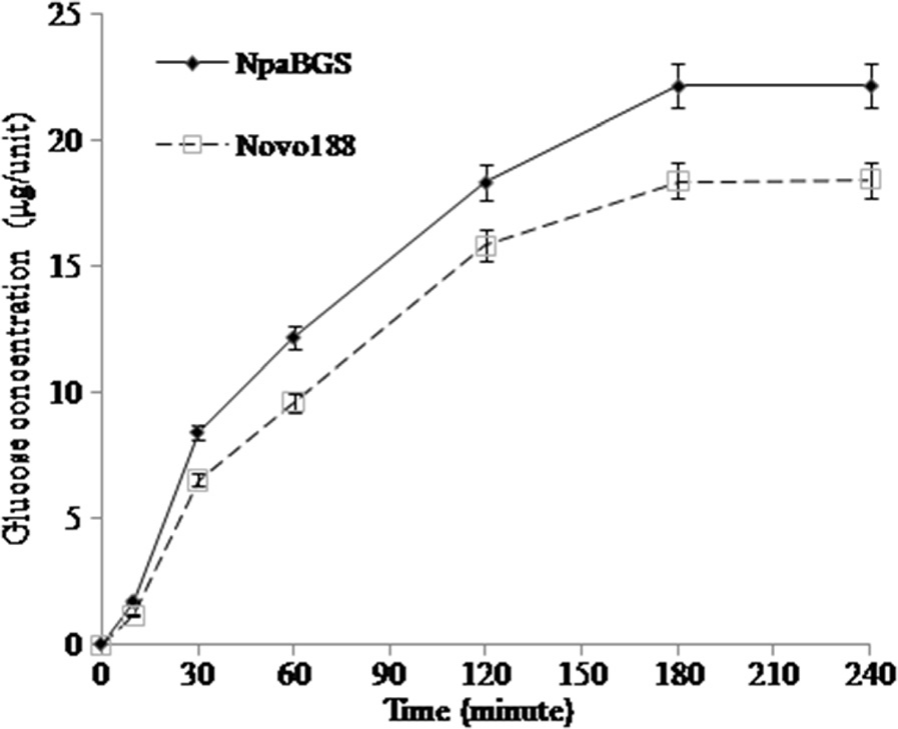
**The time course assay for cellobiose digestion by NpaBGS or Novo 188.** Both enzymatic assays were conducted by incubating each enzyme at 40°C for different durations in 100 mM in Tris–HCl buffer (pH 6.0) and then measured their β-glucosidase activity against cellobiose.

The effects of NpaNGS and Novo 188 on ethanol fermentation in SSF at different temperatures were also studied. When the yeasts were inoculated in SSF at 30°C, the yeast cultured with NpaBGS showed a better performance in ethanol conversion than the yeast cultured with Novo 188; the same results were observed for both yeast hosts KY3 and *S. cerevisiae* (Figure 4B). In addition, because 40°C is the optimal condition for ethanol production by *K. marxianus* KY3 [40] and for the enzyme reaction of NpaBGS (Figure 2), a higher ethanol productivity was observed at 40°C than at 37°C and 30°C (Figure 4B). Although the yeasts stopped producing ethanol after 12 h of culturing, more than 50 % cellobiose was converted to ethanol by *K. marxianus* KY3 in the SSF process at 37°C and 40°C. The efficiency of cellobiose digestion by either NpaBGS or Novo 188 was significantly lowered after 12 hours of reaction (data not shown), but the digested sugar was still sufficient to support a weak growth of the yeasts (Figure 4A). These data indicated that removing the feedback inhibitor (glucose) by yeasts could enhance the activity of β-glucosidase, as reflected by the ethanol productivity by both *S. cerevisiae* BY4741 and *K. marxianus* KY3. Note that the ethanol production rate at 40°C and the growth rate at 37°C of *K. marxianus* KY3 with 2 % cellobiose by the NpaBGS treatment was done using 2 % glucose as the sole carbon source (data not shown). These SSF results indicated that NpaBGS had a significantly higher efficiency for SSF ethanol production by both yeast hosts than Novo 188 at all the temperatures tested, probably due to the faster cellobiose-digestion rate of NpaBGS than Novo 188 under the temperatures tested (30-40°C). In summary, our data indicates that purified NpaBGS is active under a wide range of conditions, with the maximum activity at 40°C in the weak acid condition (pH 5.0-6.0). Moreover, with 1 unit of enzyme, NpaBGS showed a good efficiency in completely converting 2 % cellobiose to glucose within 4 hours in the optimal buffer system (Figure 5). The time course assay with the two enzymes for cellobiose digestion was also examined at 40°C and pH 6.0, and the data indicated that NpaBGS had a slightly better efficiency than Novo 188.

### **Cellulosic ethanol conversion from napiergrass**

**Figure 6:**
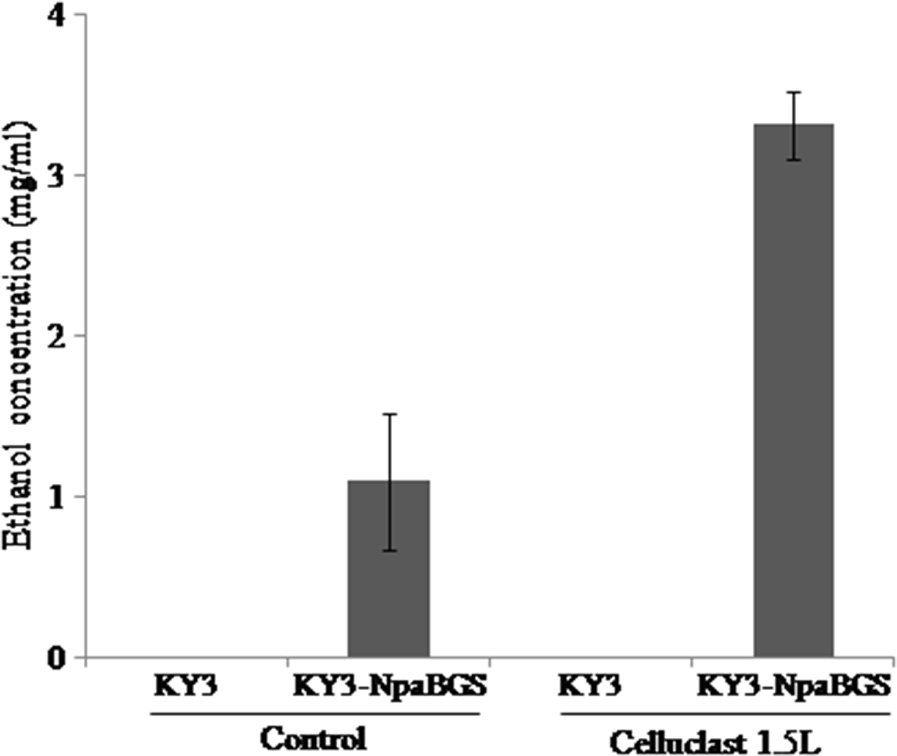
**The ethanol productivity of the SSF experiment using 2 % dry napiergrass as the solo carbon source.** A 2 ml of Celluclast 1.5 L was added to 50 ml yeast cultures and two different strains, KY3 and KY3-NpaBGS, were inoculated in one day aerobic culturing at 40°C.

In the current process of SSF, the commercial Celluclast 1.5 L is usually employed for converting cellulose to cellobiose or oligosaccharides. Although Celluclast 1.5 L has high exo- and endo-glucanase activities, it has a very low β-glucosidase activity. Therefore, it is necessary to add a β-glucosidase, such as Novo 188, to convert cellobiose to glucose. Instead of adding β-glucosidase, one may transform a β-glucosidase gene into the host. For this purpose, we succeeded in expressing the NpaBGS gene in *K. marxianus* KY3, and the new strain was employed in SSF using dry napiergrass (2 %) as the feeding stock. While the wild-type KY3 alone could not produce any detectable amount of ethanol, the engineered strain KY3-NpaBGS could produce 1.09 mg/ml ethanol, probably by using the oligosaccharides of napiergrass, such as cellobiose and cellodextrin (Figure 6). Furthermore, as a SSF experiment we added 2 ml of Celluclast 1.5 L to 50 ml yeast cultures and compared their effects on KY3 and KY3-NpaBGS alcoholic fermentation. Again, KY3 produced no detectable amount of ethanol, while the strain KY3-NpaBGS could produce 3.32 mg/ml ethanol from napiergrass in one day aerobic culturing at 40°C (Figure 6). Although the efficiency of ethanol conversion was low, as a proof of concept the experiment showed that the NpaBGS gene has a potential to be applied in SSF or even in CBP if we also introduce both exo- and endo-glucanase genes into the host. The efficiency of ethanol production of SSF can be improved by using an anaerobic system, increasing the copy number of the NpaGNS genes and immobilizing the enzymes on the cell surface.

For practical applications such as the separate hydrolysis and fermentation (SHF) process, most of the commercial cellulases show a higher efficiency and thermostability at temperatures higher than 40°C [41]. In bio-fuel industry, SSF and simultaneous saccharification and co-fermentation (SSCF) are the two major processes currently used for cellulosic-ethanol production. However, most efficient microbes for ethanol production such as brewer’s yeast live below 40°C. For highly efficient SSF or SSCF, the enzymes should have a large capacity to digest the substrates in order to provide carbon sources for the microbes to grow under the same culture condition. A previously characterized rumen cellulase showed an optimal activity at pH from 4.0 to 7.0 and temperature from 35°C to 50°C [18]. In our experiments, two alcoholic fermentation yeasts were chosen to study the performances of NpaBGS and Novo 188 in SSF at different temperatures. Cell density, glucose concentration and ethanol concentration were used to evaluate the SSF efficiency. Although Novo 188 showed better digestion efficiency and thermostability than NpaBGS at higher temperatures (Figure 2), NpaBGS appeared to be superior to Novo 188 at 40°C. Since 40°C is almost the highest temperature for microbe growth and fermentation, NpaBGS has a potential for a direct application in a bio-reactor system and for CBP.

Although the SSF concept has already been published in many previous studies, most of these studies focused on the enzyme cocktail and the enzyme/substrate blending proportion. Indeed, the related enzyme technology focused on enhancing the thermo-stability or increasing the acid tolerance of the cellulolytic enzymes. In this study, we isolated a new beta-glucosidase and showed that it has advantages over the commercial enzyme Novo 188 in SFF applications and that it can be transformed into a host to replace the use of Novo 188. Since NpaBGS was isolated from one of the best nature SSF system, the buffalo rumen, which undergoes microbial fermentation in 38°C, it may have an advantage for constructing an artificial SSF system for yeast ethanol production. We considered a friendly environment for both host growth and the enzyme reaction. This new concept has the potential to reduce the cost as it requires no external supply of beta-glucosidase and it might improve the efficiency of a bio-process, such as SSF or CBP.

## **Conclusions**

We have successfully cloned and expressed an extracellular β-glucosidase from *N. patriciarum* W5 and investigated its enzymatic properties. The optimal activity was detected at 40°C and pH 5–6, showing that the enzyme prefers a weak acid condition. Moreover, its activity at 50°C could be enhanced by adding Mg^2+^ or Mn^2+^ ions. The enzyme, NpaBGS, showed advantages in cell growth, glucose production, and ethanol production over the commercial enzyme Novo 188 in SSF. In addition, it was also employed with Celluclast 1.5 L to conduct an SSF experiment using dry napiergrass as the sole carbon source. The work presented here is the first study that considers an environment that is friendly for both host growth and the enzyme reaction. This new concept can help to improve the efficiency of bio-process, such as SSF and CBP, and it marks a notion to reassess what has missed in the old industry. Although many different fungal β-glucosidases have been identified and cloned, only a few were derived from rumen fungi. In this study, a novel β-glucosidase, NpaBGS, was isolated from buffalo rumen, which is one of the best natural SSF systems for microorganism fermentation at 38°C, and it is shown to have a good potential for an artificial SSF system for ethanol production. .

## **Methods**

### **Cloning of NpaBGS cDNA**

The cDNA of NpaBGS was amplified by PCR using the Platinum Taq DNA Polymerase High Fidelity (Invitrogen, Carlsbad, CA, USA) with the gene-specific primers flanked by restriction site sequences, EcoRI/F1-1 (AATTCATGAAGTTCTCATCTGTTTTATCTACTG), EcoRI/F1-2 (CATGAAGTTCTCATCTGTTTTATCTACTG), XhoI/R1-1 (GTTAGTAAAGTTTGTAAGCTCTCTTC), XhoI/R1-2 (TCGAGTTAGTAAA GTT TGTAAGCTCTCTTC), and PCR products were cloned into the pGEM-T vector (Promega Corp. Madison, WI, USA). The plasmid DNA from a positive clone was digested with *Xho*I and *Eco*RI and subcloned in frame with the *P. pastoris* pPICZ A expression vector. After sequencing confirmation of the NpaBGS cDNA, the recombinant plasmid, pPA-NpaBGS, was used to transform the *P. pastoris* GS115 (his4) strain (Invitrogen).

### **Construction of*Pichia pastoris*recombinant strains expressing NpaBGS**

Ten μg of the pPICZA-NpaBGS DNA was linearized with *Bgl*II and transformed into the yeast *P. pastoris* GS115 strain by electroporation. A 200 μL aliquot was spread on YPD plates containing 100 μg/mL zeocin and incubated at 30°C. Another aliquot of electroporated cells was spread onto YPD plates containing 1,000 μg/mL zeocin to screen for colonies with high copy insertion. One transformant confirmed for the Mut^+^ phenotype was scored and grown in 10 mL of BMGY medium. The yeast colony was cultured at 30°C with orbital shaking at 250 rpm for about 20 h until the density reached OD_600_ of 5.0. The yeast culture was harvested by centrifugation at 2,000 x *g* for 5 min at room temperature. To induce expression via the AOX1 promoter, the pellet was resuspended in 50 mL of BMMY medium and grown at 30°C with 250 rpm shaking for 5 days, during which methanol was added to the concentration of 0.5 % at 24-hr intervals to maintain induction, and the activity of the culture was examined simultaneously. In order to increase the level of induction, various concentrations of methanol (from 0.5 to 3 %) were also tested.

### **Purification of recombinant NpaBGS**

The yeast broth (4 L) was percolated through filter paper (Toyo Roshi Kaisha, Japan) and concentrated with a stirred ultrafiltration cell (model 8400; Millipore Corp.) equipped with a PM 10 membrane (Millipore Corp., USA) under the nitrogen pressure of 4.0 kg. f/cm^2^ and dialyzed against 20 mM sodium acetate buffer (pH 5.0). The extracted enzyme was condensated by precipitation at increasing concentrations of ammonium sulfate (0-30 %, 30-50 %, and 50-70 %) at 5°C. The fraction contained better activity and amount of enzyme was found at 50-70 % ammonium sulfate precipitation. The resulting precipitates were collected by cold centrifugation, dissolved in distilled water and dialyzed (0.1 M phosphate buffer, pH 6.0, 48 h, 5°C) to remove excessive salt. The protein (30 mL) was then loaded onto a Toyopearl DEAE-650 S (Tosoh, Japan) column (2.0× 20 cm) and eluted with a step gradient of 0, 200, 300, 400, and 500 mM of NaCl in a volume of 1,000 mL. The fractions showing cellulolytic activity were pooled and concentrated by ultrafiltration, then dialyzed against 50 mM sodium acetate (pH 5.0) containing 0.15 M NaCl. The dialyzed sample (4 mL) was applied to a Sephacryl 300-S HR (GE Healthcare Bio-Sciences AB) column (1.6 × 60 cm) and eluted with the same buffer at a flow rate of 0.5 ml/min. The active fractions were concentrated by ultrafiltration and dialyzed against 20 mM Tris–HCl buffer (pH 8.0) containing 1.5 M (NH_4_)_2_SO_4_. The dialyzed enzyme solution (2.0 mL) was then loaded onto a Resource PHE (Amersham Biosciences, USA) column (1.0 × 1.0 mL) equilibrated with the same buffer containing 1.5 M (NH_4_)_2_SO_4_. The active fractions were eluted with a decreasing linear gradient from 1.5 to 0 M of (NH_4_)_2_SO_4_ in the buffer at a flow rate of 1.0 mL/min. The fractions containing the β-glucosidase activity were further concentrated with an Ultrafree-0.5 centrifugal filter (Millipore Corp., USA), and the purity was checked by sodium dodecyl sulfate-polyacrylamide gel electrophoresis (SDS-PAGE). Protein samples were electrophoresed on 12 % (w/v) SDS–PAGE gels and visualized by staining with Coomassie Brilliant Blue R-250 [42]. The molecular weight markers were obtained from Fermentas (USA). Protein concentration was determined using a protein assay kit (Bio-Rad Laboratories Inc., Hercules, California, USA). Native PAGE electrophoresis was carried out similarly by exclusion of SDS from all solutions. Zymogram after native PAGE was performed as described by Feng et al. [43] and Duan et al. [18].

### **Characterization of recombinant NpaBGS enzymatic properties**

After optimizing the culture conditions of the *P. pastoris* recombinant strain for producing NpaBGS, the β-glucosidase activity in the culture medium supernatant was measured. β-glucosidase activity was assayed by adding 10 μl of culture supernatant to 90 μL of 2 % (w ⁄ v) cellobiose (low viscosity), and the amount of glucose assay was carried out according to the manual of Glucose (HK) assay kit (Sigma-Aldrich, USA). One unit (U) of β-glucosidase activity was defined as the amount of 1 μmole glucose released per minute at pH 7.0 under the assay conditions described below. Furthermore, other natural substrates, such as amygdalin, arbutin, larminarin, phenyl-β-D-glucoside, and β-gentiobiose, and substrates, such as 4-methylumbel-liferyl-β-D-cellobioside (MUC), 4-mehtylumbelliferyl-β-D-galactopyronoside (MUG) and 4-methylumbel-liferyl-β-D- glucopyranoside (MUD) were also assayed at in the same condition.

The temperature profile and the optimum temperature of NpaBGS were determined by the activity on cellobiose at 30, 40, 50, and 60°C. The optimum pH was determined using 100 mM buffers: sodium citrate (pH 2.0 and 3.0), sodium acetate (from pH 4.0 to pH 6.0) and Tris–HCl (form pH 6 to pH 9). The thermostability of NpaBGS and the commercial enzyme Novo™ 188 (Novozymes, Bagsvaerd, Denmark) were compared by incubation at 40°C in 100 mM in Tris–HCl buffer (pH 6.0) during a time course and then measured their β-glucosidase activity against cellobiose.

To evaluate the effect on the β-glucosidase activity of metal cations, such as Al^3+^, Ca^2+^, Cu^2+^, Fe^3+^, Mg^2+^, Mn^2+^, and Zn^2+^, and reducing agents, such as DTT, and β-mercaptoenthanol, these elements were added separately to the standard assay in 100 mM Tris–HCl buffer, pH 6.0, to a final concentration of 1 and 10 mM, respectively.

### **Efficiency of β-glucosidase in SSF application**

*S. cerevisiae* BY4741 and *Kluyveromyces marxianus* KY3 [40] were used in SSF experiments to study the effect of different culture temperatures. Both yeasts could grow at 30°C on the solid YPAD medium (1 % yeast extract, 2 % peptone, 24 mg/L adenine hemisulfate, 2 % glucose, and 2 % agar), while separate cultures of *K. marxianus* KY3 was also examined at 37°C and 40°C for its thermotolerance. First, the two microbes were pre-cultured overnight in YPAD medium and inoculated to initial O.D._600nm_ 0.1 in 10 ml of fresh YPA medium containing either 2 % glucose or 2 % cellobiose under an aerobic condition. The experimental groups were the YPA medium with 2 % cellobiose and supplemented with an equal unit (2 units) of NpaBGS or Novo™ 188 β-glucosidase in the culture. The growth curve, glucose generation and ethanol production of these cultures were determined at 0, 12, 16, 20, and 24 hrs. The cell density was measured at 600 nm by spectrophotometer (Ultrospec 2100 pro, GE Healthcare Bio-Sciences AB). In a parallel experiment, the SSF experiment was conducted using 2 % dry napiergrass as the sole carbon source, and adding 2 ml of commercial Celluclast 1.5 L in a 50 ml yeast culture at 40°C. In addition, *K. marxianus* KY3 and KY3-NpaBGS, in which KY3 was transformed with the NpaBGS gene via a commercial expression system (*K. lactis* Protein Expression Kit, New England Biolabs) [40], were used in the SSF experiment. Glucose and ethanol assays were performed using Glucose (HK) assay kits and Ethanol Enzymatic BioAnalysis kits (Roche Molecular Biochemicals, Germany) following manufacturer’s procedures.

## **Abbreviations**

CMC = Carboxymethyl cellulose; DTT = Dithiothreitol; MUC = 4-methylumbel-liferyl-β-D-cellobioside; MUD = 4-methylumbel-liferyl-β-D-glucopyranoside; MUG = 4-mehtylumbelliferyl-β-D-galactopyronoside; SSF = Simultaneous saccharification and fermentation.

## **Competing interests**

The authors declare that they have no competing interests.

## **Authors’ contributions**

H-L C, Y-C C, J-J C and M-Y J L designed experiments. H-L C, M-Y J L, J-J C, H-M K, S-K R, T-Y W, K-Y H, H-Y C, and W-T L carried out the experiments, analyzed the data and drafted the manuscript. H-L C, J-J C and T-Y W drafted the manuscript. M-C S and W-H L supervised the study and revised the manuscript. All authors read and approved the final manuscript.

## Supplementary Material

Additional file 1 Nucleotide and deduced amino acid sequences of the NpaBGS cDNA. Amino acids are represented below the nucleotide sequence. The signal peptide is labeled with asterisks. The potential sites of *N*-glycosylation and activity site are shown in gothic font. The predicted domains of GH3 at the N and C terminal are underlined. Click here for file

Additional file 2 NpaBGS enzyme activity on CMC. Click here for file
