# Supplementary figures and images for: A highly efficient β-glucosidase from the buffalo rumen fungus Neocallimastix patriciarum W5
Source: Biotechnol Biofuels. 2012 Apr 19;5:24. doi: 10.1186/1754-6834-5-24 (PMC3403894; doi:10.1186/1754-6834-5-24)

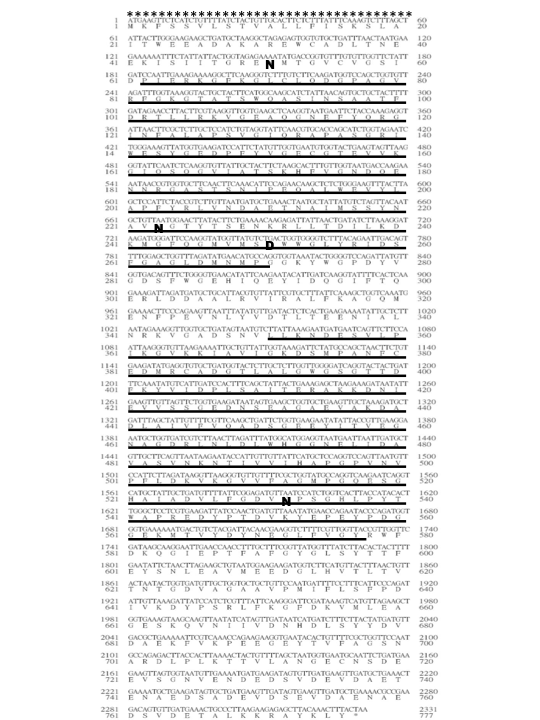

Supplement: Additional file 1 — Nucleotide and deduced amino acid sequences of the NpaBGS cDNA. Amino acids are represented below the nucleotide sequence. The signal peptide is labeled with asterisks. The potential sites of N-glycosylation and activity site are shown in gothic font. The predicted domains of GH3 at the N and C terminal are underlined. [file 1754-6834-5-24-S1.tiff]
