# Supplementary material for: A highly efficient β-glucosidase from the buffalo rumen fungus Neocallimastix patriciarum W5
Source: Biotechnol Biofuels. 2012 Apr 19;5:24. doi: 10.1186/1754-6834-5-24 (PMC3403894; doi:10.1186/1754-6834-5-24)
Supplement: Additional file 2 — NpaBGS enzyme activity on CMC. [file 1754-6834-5-24-S2.pdf]

NpaBGS enzyme activity on CMC

Method: The endo-glucosidase activity of NpaBGS was assayed by adding 10 μl of purified enzyme to 90 μl of 2 % (w/v) CMC (Sigma, U.S.A.), and incubated at 40°C for 4hr. A commercial bench maker (EGIII, Megazyme, Ireland) were diluted to 1/400, 1/800, 1/1600, 1/3200, and 1/6400 as a standard in evaluating the NpaBGS activity. Then all the samples were tested by the Somogyi-Nelsonmethod(S-N assay) to detect the endo-glucosidase activity (triplicates). The micro-assay for reduced sugars was a modification of the S-N assay. In a 96-well microplate, 25 μl of sample and 25 μl of appropriate substrate solubilized in 0.1 M citrate buffer, pH 5.0, were placed in each well. The plate was covered with an acetate adhesive sheet and incubated at 40°C for 24 h. After incubation 75 μl of Somogyi copper reagent was added, and the wells were resealed and placed in a water bathat80°C for 30 min. After the plate had cooled completely (~ 15 min), 75 μl of arsenomolybdate was added, and the wells were mixed well with a Vortex mixer. Colorimetric measurements were read using reflectance at 520 nm with a spectrophotometer (Anthos Zenyth 200rt, Biochrom Limited, England).

Results: After the measurement by a spectrophotometer at 520 nm, the values were 0.64±0.04, 0.56±0.02, 0.43±0.03, and 0.32±0.05 for commercial EGIII as the bench marker that was diluted to1/400, 1/800, 1/1600, 1/3200, and 1/6400, respectively. The NpaBGS was put in the same condition to evaluate if it had endo-glucosidase activity. The value of NpaBGS was 0.46±0.02, and was equal to the diluted EGIII in 1/1600. Although NpaBGS showed a major activity of β–glucosidase, it had weak endo-glucosidase activity to digest the linear chain of cellulose, such as CMC. It suggested that the dual function of NpaBGS makes it efficient to digest the native CMC into glucose.

References:

Somogyi, M. (1952). Notes on sugar determination. J. Biol. Chem. 195:19-23.

Nelson, N. (1994). A photometric adaptation of the Somogyi method for the determination of glucose J. Biol. Chem. 195:19-23.
